# Supplementary material for: Trends in using intraoperative parathyroid hormone monitoring during parathyroidectomy: Protocol and rationale for a cross-sectional survey study of North American surgeons
Source: PLoS One. 2024 Jul 9;19(7):e0301153. doi: 10.1371/journal.pone.0301153 (PMC11233005; doi:10.1371/journal.pone.0301153)
Supplement: S1 Appendix — (DOCX) [file pone.0301153.s001.docx]

**S1 Appendix.** Survey cover letter and questionnaire

**Title**: Trends in using intraoperative parathyroid hormone monitoring during parathyroidectomy: Protocol and rationale for a cross-sectional survey study of North American surgeons.

**Principal Investigator:** Dr. Han Zhang, MD FRCSC
**Affiliation:** Division of Otolaryngology­­–Head and Neck Surgery, Department of Surgery, McMaster University
**Email address:** [hanzhang@stjosham.ca](mailto:hanzhang@stjosham.ca)

**Funding:** None

**Invitation to Participate:**  You are invited to participate in a research study conducted by Dr. Han Zhang and Dr. Phillip Staibano.

**Purpose**: This study aims to better understand clinical decision-making regarding the use of intraoperative parathyroid hormone monitoring during parathyroid surgery.

**Participation and Withdrawal:** You are being asked to complete a brief online questionnaire that will take 10–15 minutes to complete. If you decide to participate, you can withdraw at any time by closing your browser. In addition, you may choose not to answer any, or some of the questions and remain in the study. It is not possible to withdraw from the study after completing and submitting the survey as it is anonymous. Participation is voluntary. Please include a statement that choosing to participate in the study or not will in no way affect their academic or employment standings.

**Risks**: The primary risk of this study is privacy breach, which is minimized by the anonymity of your responses.

**Benefits**: Your participation in this study will improve understanding of clinical decision-making regarding the employment of intraoperative parathyroid hormone monitoring in parathyroid surgery. There are no medical benefits for participating in study.

**Confidentiality and Privacy**: All survey responses will be used exclusively for the purposes of medical education research. We do not have access to your contact information. All data is anonymous and will be analyzed and presented in an aggregate format. If the results of the study are published, your name will not be used and no information that discloses your identity will be released or published without your specific consent to the disclosure.

**Conservation of Data**: The data collected (survey datasets) will be securely stored in Single- Sign-On protected Qualtrics and password-protected OneDrive accounts. All data will be stored on password-protected encrypted personal computers over secure networks. Data will be destroyed two years following the completion of the study.

By clicking on the link to the survey and submitting the survey, consent to this survey is implied.

This study has been reviewed by the Hamilton Integrated Research Ethics Board (HIREB). The HIREB is responsible for ensuring that participants are informed of the risks associated with the research, and that participants are free to decide if participation is right for them. If you have any questions about your rights as a research participant, please call the Office of the Chair, Hamilton Integrated Research Ethics Board at 905.521.2100 x 42013.

For the purposes of ensuring proper monitoring of the research study, it is possible that representatives of the Hamilton Integrated REB (HiREB), this institution, and affiliated sites or regulatory authorities may consult your original (non-identifiable) research data and medical records to check that the information collected for the study is correct and follows proper laws and guidelines. By participating in this study, you authorize such access.

**If you have any questions about the study, you may contact the research team at staibapm@mcmaster.ca.**

**Domain 1:** Respondent demographic and training details

Q1. What is your current age?

o > 70 years old

o 60–69 years old

o 50-59 years old

o 40-49 years old

o 30–39 years old

o 20–29 years old

Q2. What is your gender?

o Male

o Female

o Non-binary / third gender

o Prefer not to say

Q3. Please state your country of surgical practice:

o USA. Please name state:

o Canada. Please name province:

o Other:

Q4. What is your current level of surgical training?

o Practicing surgeon

o Clinical fellow

o Senior resident (PGY 3–5)

o Junior resident (PGY 1-2)

o Other:

Q5. Please describe your type of surgical practice:

o Only academic

o Only community

o Mixed academic and community. Please describe percentage (0-100%):

o Other:

Q6. How many years have you been in practice?

o 30 years

o 20-29 years

o 10-19 years

o 1-9 years

o N/A: I am a resident/fellow.

Q7. Please describe your residency training:

o General surgery residency. Please name institution:

o Otolaryngology–Head and Neck Surgery. Please name institution:

o Other:

Q8. Did you perform parathyroid surgery during your residency training?

o Never

o Rarely

o Sometimes

o Most of the time

o Always

Q9. Did you utilize intraoperative parathyroid hormone monitoring during your surgical training?

o Yes, I used it in both residency and fellowship

o Yes, but only in residency training

o Yes, but only in fellowship training

o No, I did not use intraoperative parathyroid hormone monitoring during my surgical training.

Q10. If you did complete a surgical fellowship, please describe your fellowship training:

o I completed fellowship in head & neck surgical oncology. Please name institution:

o I completed fellowship in endocrine surgery. Please name institution:

o I completed a fellowship in surgical oncology. Please name institution:

o Other. Please describe:

o N/A: I did not complete a fellowship.

Q11. Do you train surgical fellows in your practice?

o No

o Maybe

o Yes

Q12. Please describe the main scope of your surgical practice:

o General OHNS

o Head and neck oncology

o General surgery

o Endocrine surgery

o Other. Please describe:

**Domain 2:** Surgical adjuncts during parathyroid surgery

Q13. How many parathyroid surgeries do you perform per year in your surgical practice?

o >50

o 40-49

o 30-3

o 20-29

o 10-19

o 0-9

o Other. Please describe:

Q14. What type of imaging do you acquire before parathyroidectomy for primary hyperparathyroidism (Please select all that apply)?

o PET-guided MRI and/or CT

o CT (4D or parathyroid protocol)

o Surgeon-performed ultrasound

o Radiologist-performed ultrasound

o Parathyroid scintigraphy

o None

o Other. Please describe:

o I do not perform surgery in this patient population.

Q15. What type of imaging do you acquire before parathyroidectomy for secondary hyperparathyroidism (Please select all that apply)?

o PET-guided MRI and/or CT

o CT (4D or parathyroid protocol)

o Surgeon-performed ultrasound

o Radiologist-performed ultrasound

o Parathyroid scintigraphy

o None

o Other. Please describe:

o I do not perform surgery in this patient population.

Q16. What type of imaging do you acquire before parathyroidectomy for tertiary hyperparathyroidism (Please select all that apply)?

o PET-guided MRI or CT

o CT (4D or parathyroid protocol)

o Surgeon-performed ultrasound

o Radiologist-performed ultrasound

o Parathyroid scintigraph

o None

o Other. Please describe:

o I do not perform surgery in this patient population.

Q17. How important do you think intraoperative parathyroid hormone is for guiding parathyroidectomy for primary hyperparathyroidism?

o Not at all important

o Slightly important

o Moderately important

o Very important

o Extremely important

Q18. How important do you think intraoperative parathyroid hormone is for guiding parathyroidectomy for secondary hyperparathyroidism?

o Not at all important

o Slightly important

o Moderately important

o Very important

o Extremely important

Q19. How important do you think intraoperative parathyroid hormone is for guiding parathyroidectomy for tertiary hyperparathyroidism?

o Not at all important

o Slightly important

o Moderately important

o Very important

o Extremely important

Q20. Does your institution use intraoperative parathyroid hormone monitoring for parathyroidectomy?

o No

o Yes

o Other. Please describe:

Skip To: Q28 If Does your institution use intraoperative parathyroid hormone monitoring for parathyroidectomy? = No

Q21. How long has your institution been using intraoperative parathyroid hormone monitoring?

o < 1 year

o 1-5 years

o 6-10 years

o 11-15 years

o 16-20 years

o > 20 years

Q22. Does your institution employ point-of-care or central laboratory analysis for intraoperative parathyroid hormone monitoring?

o Point-of-care instrument

o Central laboratory instrument

o Other. Please describe:

Q23. Please estimate your sample turnaround time (TAT) from time of sample draw to result reporting:

o 0-5 minutes

o 6-10 minutes

o 11-15 minutes

o 16-20 minutes

o 21-25 minutes

o 26-30 minutes

o > 30 minutes. Please estimate:

Q24. Please indicate that intraoperative parathyroid monitoring criteria that you use to guide surgery for primary hyperparathyroidism (Please check all that apply):

o Miami/modified Miami criteria: A >50% ioPTH drop from the highest either pre-incision or pre-excision at 10 or 15 minutes after excision of all hyperfunctioning parathyroid gland(s).

o Vienna criteria: A >50% ioPTH drop from the pre-incision value within 10 minutes after excision of all hyperfunctioning parathyroid gland(s).

o Rome criteria: A >50% ioPTH drop from highest pre-excision level and/or ioPTH level within normal range at 20 minutes post-excision, and/or ≤0.80 pmol/L less than the value at 10 minutes post-excision.

o Halle criteria: An ioPTH decay into the low normal range (< 35 ng/L) within 15 minutes of removing all hyperfunctioning parathyroid glands.

o Other. Please name and describe parameters:

Q25 Please indicate your preferred baseline sampling intervals for intraoperative parathyroid hormone monitoring (If you perform more than one baseline sample, please check all that apply):

o Before general anesthetic

o Before surgical skin incision, but after general anesthetic

o Between skin incision and ligation of first parathyroid gland

o Other. Please describe:

Q26. Please indicate your preferred post-gland ligation sampling intervals for intraoperative parathyroid hormone monitoring (If you perform more than one post-gland ligation sample, please check all that apply):

o 5 minutes post-parathyroid gland ligation

o 10 minutes post-parathyroid gland ligation

o 15 minutes post-parathyroid gland ligation

o 20 minutes post-parathyroid gland ligation

o 25 minutes post-parathyroid gland ligation

o Other. Please describe:

Q27. Please indicate your preferred venous sampling technique for intraoperative parathyroid hormone monitoring:

o Peripheral intravenous line. Please indicate location:

o Peripheral arterial line

o Central venous draw (i.e., Internal jugular vein)

o Other. Please describe:

Q28. Please name any intraoperative surgical adjuncts that you employ during parathyroidectomy (If you use more than one type of surgical adjunct, please check all that apply):

o Intraoperative frozen section analysis

o Intraoperative parathyroid hormone monitoring

o I do not use any intraoperative surgical adjuncts during parathyroidectomy

o Other. Please describe:

Display This Question:

If “Does your institution use intraoperative parathyroid hormone monitoring for parathyroidectomy?” = No

Q29. Please estimate the number of years before you believe your institution will acquire intraoperative parathyroid hormone monitoring technology:

o <1 year

o 1-5 years

o 6-10 years

o >11 years

o Other. Please describe:

Display This Question:

If “Does your institution use intraoperative parathyroid hormone monitoring for parathyroidectomy?” = No

Q30. Please list perceived barriers to acquisition of intraoperative parathyroid hormone monitoring technology at your institution (Please check all that apply):

o Clinical barriers (e.g., differing surgeon opinion regarding utility of this technology). Please describe:

o Economic barriers (e.g., hospital unwilling to purchase). Please describe:

o Institutional barriers (e.g., lack of laboratory space, staffing). Please describe:

o Other. Please describe:

**Thank you for completing this survey and participating in this study.**
