# Supplementary material for: Trends in using intraoperative parathyroid hormone monitoring during parathyroidectomy: Protocol and rationale for a cross-sectional survey study of North American surgeons
Source: PLoS One. 2024 Jul 9;19(7):e0301153. doi: 10.1371/journal.pone.0301153 (PMC11233005; doi:10.1371/journal.pone.0301153)
Supplement: Attachment — Submitted filename: Response_to_reviewers_PONE-D-24-07939.docx [file pone.0301153.s002.docx]

PONE-D-24-07939
Trends in using intraoperative parathyroid monitoring during parathyroidectomy: Protocol and rationale for a cross-sectional survey study of North American surgeons
PLOS ONE

Dear Dr. Staibano,

Thank you for submitting your manuscript to PLOS ONE. After careful consideration, we feel that it has merit but does not fully meet PLOS ONE’s publication criteria as it currently stands. Therefore, we invite you to submit a revised version of the manuscript that addresses the points raised during the review process.

Please provide a point-by-point response to reviewers #2 and #3. Unfortunately reviewer #1 assessed the manuscript as a final paper and not as a protocol. Looking forward to your revised manuscript.

Please submit your revised manuscript by Jul 26 2024 11:59PM. If you will need more time than this to complete your revisions, please reply to this message or contact the journal office at [plosone@plos.org](mailto:plosone@plos.org). When you&apos;re ready to submit your revision, log on to <https://www.editorialmanager.com/pone/> and select the &apos;Submissions Needing Revision&apos; folder to locate your manuscript file.

Please include the following items when submitting your revised manuscript:

- A rebuttal letter that responds to each point raised by the academic editor and reviewer(s). You should upload this letter as a separate file labeled &apos;Response to Reviewers&apos;.
- A marked-up copy of your manuscript that highlights changes made to the original version. You should upload this as a separate file labeled &apos;Revised Manuscript with Track Changes&apos;.
- An unmarked version of your revised paper without tracked changes. You should upload this as a separate file labeled &apos;Manuscript&apos;.

If you would like to make changes to your financial disclosure, please include your updated statement in your cover letter. Guidelines for resubmitting your figure files are available below the reviewer comments at the end of this letter.

If applicable, we recommend that you deposit your laboratory protocols in <protocols.io> to enhance the reproducibility of your results. <Protocols.io> assigns your protocol its own identifier (DOI) so that it can be cited independently in the future. For instructions see: <https://journals.plos.org/plosone/s/submission-guidelines#loc-laboratory-protocols>. Additionally, PLOS ONE offers an option for publishing peer-reviewed Lab Protocol articles, which describe protocols hosted on <protocols.io>. Read more information on sharing protocols at <https://plos.org/protocols?utm_medium=editorial-email&utm_source=authorletters&utm_campaign=protocols>.

We look forward to receiving your revised manuscript.

Kind regards,

Mabel Aoun, MD, MPH
Academic Editor
PLOS ONE

Journal Requirements:

**1. When submitting your revision, we need you to address these additional requirements.

Please ensure that your manuscript meets PLOS ONE&apos;s style requirements, including those for file naming. The PLOS ONE style templates can be found at**
[**https://journals.plos.org/plosone/s/file?id=wjVg/PLOSOne_formatting_sample_main_body.pdf**](https://journals.plos.org/plosone/s/file?id=wjVg/PLOSOne_formatting_sample_main_body.pdf) **and**
[**https://journals.plos.org/plosone/s/file?id=ba62/PLOSOne_formatting_sample_title_authors_affiliations.pdf**](https://journals.plos.org/plosone/s/file?id=ba62/PLOSOne_formatting_sample_title_authors_affiliations.pdf)**.**

Thank you – we did adjust style and file naming to meet PLOS ONE requirements.

**2. When completing the data availability statement of the submission form, you indicated that you will make your data available on acceptance. We strongly recommend all authors decide on a data sharing plan before acceptance, as the process can be lengthy and hold up publication timelines. Please note that, though access restrictions are acceptable now, your entire data will need to be made freely accessible if your manuscript is accepted for publication. This policy applies to all data except where public deposition would breach compliance with the protocol approved by your research ethics board. If you are unable to adhere to our open data policy, please kindly revise your statement to explain your reasoning and we will seek the editor&apos;s input on an exemption. Please be assured that, once you have provided your new statement, the assessment of your exemption will not hold up the peer review process.**

We have made all associated documents available. Since this is a protocol, there is no collected data that requires sharing. All authors agree to full data sharing plan.

**3. Ethics statement appears in the Methods section of the manuscript AND at the end of the manuscript:
Your ethics statement should only appear in the Methods section of your manuscript. If your ethics statement is written in any section besides the Methods, please delete it from any other section.**

We have made sure to only include this in the methods section.

**4. Please include captions for your Supporting Information files at the end of your manuscript, and update any in-text citations to match accordingly. Please see our Supporting Information guidelines for more information:** [**http://journals.plos.org/plosone/s/supporting-information**](http://journals.plos.org/plosone/s/supporting-information)**.**

We did change this match formatting guidelines.

**5. Please review your reference list to ensure that it is complete and correct. If you have cited papers that have been retracted, please include the rationale for doing so in the manuscript text, or remove these references and replace them with relevant current references. Any changes to the reference list should be mentioned in the rebuttal letter that accompanies your revised manuscript. If you need to cite a retracted article, indicate the article’s retracted status in the References list and also include a citation and full reference for the retraction notice.**

All references have been reviewed.

**Comments to the Author**

1. Does the manuscript provide a valid rationale for the proposed study, with clearly identified and justified research questions?

The research question outlined is expected to address a valid academic problem or topic and contribute to the base of knowledge in the field.

Reviewer #1: No

Reviewer #2: Partly

Reviewer #3: Yes

2. Is the protocol technically sound and planned in a manner that will lead to a meaningful outcome and allow testing the stated hypotheses?

The manuscript should describe the methods in sufficient detail to prevent undisclosed flexibility in the experimental procedure or analysis pipeline, including sufficient outcome-neutral conditions (e.g. necessary controls, absence of floor or ceiling effects) to test the proposed hypotheses and a statistical power analysis where applicable. As there may be aspects of the methodology and analysis which can only be refined once the work is undertaken, authors should outline potential assumptions and explicitly describe what aspects of the proposed analyses, if any, are exploratory.

Reviewer #1: No

Reviewer #2: Yes

Reviewer #3: Yes

3. Is the methodology feasible and described in sufficient detail to allow the work to be replicable?

Descriptions of methods and materials in the protocol should be reported in sufficient detail for another researcher to reproduce all experiments and analyses. The protocol should describe the appropriate controls, sample size calculations, and replication needed to ensure that the data are robust and reproducible.

Reviewer #1: No

Reviewer #2: Yes

Reviewer #3: Yes

4. Have the authors described where all data underlying the findings will be made available when the study is complete?

The [PLOS Data policy](https://journals.plos.org/plosone/s/materials-and-software-sharing) requires authors to make all data underlying the findings described in their manuscript fully available without restriction, with rare exception, at the time of publication. The data should be provided as part of the manuscript or its supporting information, or deposited to a public repository. For example, in addition to summary statistics, the data points behind means, medians and variance measures should be available. If there are restrictions on publicly sharing data—e.g. participant privacy or use of data from a third party—those must be specified.

Reviewer #1: No

Reviewer #2: Yes

Reviewer #3: No

5. Is the manuscript presented in an intelligible fashion and written in standard English?

PLOS ONE does not copyedit accepted manuscripts, so the language in submitted articles must be clear, correct, and unambiguous. Any typographical or grammatical errors should be corrected at revision, so please note any specific errors here.

Reviewer #1: No

Reviewer #2: Yes

Reviewer #3: Yes

6. Review Comments to the Author

Please use the space provided to explain your answers to the questions above and, if applicable, provide comments about issues authors must address before this protocol can be accepted for publication. You may also include additional comments for the author, including concerns about research or publication ethics.

You may also provide optional suggestions and comments to authors that they might find helpful in planning their study.

(Please upload your review as an attachment if it exceeds 20,000 characters)

**Reviewer #1: Thank you for giving me the opportunity to review this manuscript.
This manuscript did not include any study outcomes. I could not evaluate the importance of this manuscript without results. Please re-submit after finalizing the study.**

Thank you for taking the time to review this manuscript.

**Reviewer #2: In this original manuscript by Staibano et al., authors report the protocole of a survey they plan to start on March, 2024 about the use of intra-operative parathyroid hormone (IOPTH) measurement during parathyroidectomy either for primary, secondary, or tertiary hyperparathyroidism. IOPTH is being more and more used for primary hyperparathyroidism (PHPT) but very few is known about its use when treating secondary (SHPT) or tertiary (THPT) hyperparathyroidism. In such, this study will help the community get a better understanding of both its use and barriers. Manuscript is overall well written and protocole is very straightforward. I still have some comments/questions authors may address.**

Thank you for taking the time to review our manuscript.

A. MAJOR COMMENTS
**1. Within the introduction section, authors suggest that "all [forms of HPT] are definitively managed via surgical extirpation". Later, they suggest that SHPT may be managed medically. So, that first statement may be more tempered: the price for "curing" SHPT may sometimes be hypoparathyroidism. Moreover, more and more data suggest that THPT may recover several months/years after kidney transplantation without surgery.**

Thank you for this comment. In the abstract and introduction sections, we did soften this language.

**2. Authors suggest that SHPT and THPT only occur during chronic kidney disease (CKD) which, if probably frequent, is not totally true: SHPT is even more frequent during vitamin D deficiency or obesity. Here, surgery should never be performed. Moreover, authors suggest that THPT may only be diagnosed after kidney transplant which, again, if frequent, is not the sole circumstance for diagnosis, dialysis being probably even more frequent.**

Thank you for this comment. In the introduction section, we did soften this language.

B. MINOR COMMENTS
**1. In the description of the study, authors mention the survey will be distributed for 6 months. A few sentences later, they state it will take place from March 28 to August 31, meaning 5 months. I&apos;d suggest being consistent between those two statements.**

To account for delayed processing in the approval of this survey by the professional societies that will oversee distribution, we have updated the time window of survey distribution.

We have clarified that survey distribution will happen from July 1 to November 1, 2024 and that the study will be completed by December 31, 2024.

**2. Regarding ethics, authors mention their survey will be &apos;anonymous&apos;. Anonymous data are data that cannot allow identifying people whom data have been collected. In the present study, crossing demographics and educational data may allow to identify participants. Therefore, such dataset is made of pseudonymous data in which no data allow direct identification. I&apos;d strongly suggest to use this wording (pseudonymous) instead of &apos;anonymous&apos; when describing such a dataset.**

Thank you – this is a very helpful comment and we have amended this to pseudonymous.

**3. In the appendix, the letter mentions that data will be stored for 5 years after the completion of the study while the manuscript states it will be for 2 years. I&apos;d suggest being consistent between those two statements.**

We did change this to two years on both documents. We also removed the survey link since this leads to a beta version of the survey that we used for validation within our team. The actual link will be sent with the email to potential respondents.

**Reviewer #3: In this paper for a protocol for a survey study of surgeons performing parathyroidectomy is presented. The study design is well presented and the protocol easy to follow.**

Thank you for your comments.

The authors should specify how they will follow the PLOS Data policy - The PLOS Data policy requires authors to make all data underlying the findings described in their manuscript fully available without restriction, with rare exception, at the time of publication. The data should be provided as part of the manuscript or its supporting information, or deposited to a public repository. For example, in addition to summary statistics, the data points behind means, medians and variance measures should be available. If there are restrictions on publicly sharing data—e.g. participant privacy or use of data from a third party—those must be specified.

7. PLOS authors have the option to publish the peer review history of their article ([what does this mean?](https://journals.plos.org/plosone/s/editorial-and-peer-review-process#loc-peer-review-history)). If published, this will include your full peer review and any attached files.

If you choose “no”, your identity will remain anonymous but your review may still be made public.

**Do you want your identity to be public for this peer review?** For information about this choice, including consent withdrawal, please see our [Privacy Policy](https://www.plos.org/privacy-policy).

Reviewer #1: **Yes:** Takahisa Hiramitsu

Reviewer #2: **Yes:** Jean-Philippe Bertocchio

Reviewer #3: No
